# Sonic hedgehog signalling regulates the self‐renewal and proliferation of skin‐derived precursor cells in mice

**DOI:** 10.1111/cpr.12500

**Published:** 2018-08-27

**Authors:** Sangkyu Park, Hyewon Kim, Kichul Kim, Sangho Roh

**Affiliations:** ^1^ Cellular Reprogramming and Embryo Biotechnology Laboratory Dental Research Institute, BK21, Seoul National University School of Dentistry Seoul Korea

## Abstract

**Objectives:**

The sonic hedgehog (Shh) signalling pathway has an important role in the maintenance of various stem cells and organogenesis during development. However, the effect of Shh in skin‐derived precursors (SKPs), which have the capacity for multipotency and self‐renewal, is not yet clear. The present study investigated the effects of the Shh signalling pathway on the proliferation and self‐renewal of murine SKPs (mSKPs).

**Methods:**

The Shh signalling pathway was activated by treatment with purmorphamine (Shh agonist) or recombinant Shh in mSKPs. Cyclopamine (Shh antagonist) or GANT‐61 (Gli inhibitor) was used to inhibit the pathway. Western blot, qPCR, and immunofluorescence were used to analyse the expression of genes related to self‐renewal, stemness, epithelial‐mesenchymal transition (EMT) and the Shh signalling pathway. In addition, cell proliferation and apoptosis were examined.

**Results:**

Inhibiting the Shh signalling pathway reduced mSKP proliferation and sphere formation, but increased apoptosis. Activating this signalling pathway produced opposite results. The Shh signalling pathway also controlled the EMT phenotype in mSKPs. Moreover, purmorphamine recovered the self‐renewal and proliferation of aged mSKPs.

**Conclusion:**

Our results suggest that the Shh signalling pathway has an important role in the proliferation, self‐renewal and apoptosis of mSKPs. These findings also provide a better understanding of the cellular mechanisms underlying SKP self‐renewal and apoptosis that allow more efficient expansion of SKPs.

## 1 INTRODUCTION

Skin‐derived precursor cells (SKPs) exist in foetal, neonatal and adult skin. SKPs are multipotent stem cells which contain various stem cell populations, including neural crest stem cells (NCSCs).^1^, ^2^, ^3^ SKPs have the potential to differentiate along various lineages. They can become adipogenic, osteogenic and chondrogenic cells. They can also become neural cells such as neurons, glial cells, and Schwann cells.^1^, ^2^, ^3^, ^4^ It has recently been shown that SKPs (like other dermal stem cells) can act in the recovery of skin damage, in wound healing and in the regeneration of hair follicles.^5^

Neural crest stem cells persist through foetal development and keep their multipotency in various parts of the body. These cells can be isolated more easily from skin than from other tissues.^6^, ^7^ SKPs exhibit many neural crest cell properties, and a number of marker genes for primitive embryonic NCSCs are also expressed in cultured SKPs.^3^, ^8^ In addition, SKP cell behaviour is similar to that of NCSCs when transplanted into the neural crest migratory stream of embryonic chicks.^3^ Although the isolation and culture of SKPs have been reported in many species (including human, rat, mouse and pig^2^, ^9^, ^10^, ^11^), the critical signalling pathways for cell property maintenance, self‐renewal and proliferation are unclear. Stem cells generally have two signatures: self‐renewal and differentiation potency. These two features in SKPs are regulated by intrinsic and extrinsic signals from various niches.^6^, ^12^

Sphere‐type SKPs are generated using a suspension culture system. Dissociated single cells from primary spheres form secondary spheres expressing the SKP markers. Various studies have reported that SKP spheres can be obtained using 3D colony‐forming systems (eg, methylcellulose or Matrigel), where the clonality of the spheres can also be confirmed.^1^, ^2^, ^13^

The Hedgehog (Hh)‐Gli signalling pathway participates in brain development, self‐renewal of neural stem cells and proliferation of various precursor cells.^14^, ^15^, ^16^ Recent reports also show that Hh‐Gli signalling pathway controls the self‐renewal of neural stem cells by regulating Nanog, p53 and Foxm1.^17^, ^18^ Hh ligands (secreted glycoproteins) bind to a cell‐surface receptor called Patched. The binding relieves its inhibition of Smoothened (Smo) and allows the signalling pathway to proceed. Smo activates the canonical Hh pathway through the Gli‐dependent transcription of multiple targets, including N‐myc, cyclin D, Patched, Gli1 and Gli2.^19^ Furthermore, the Hh signalling pathway plays a critical role in endoderm and mesoderm development during embryogenesis.^20^ Sonic hedgehog (Shh) knock‐out mice are embryonic lethal because these mice have problems patterning vertebrate embryonic tissues (including the brain, spinal cord and axial skeleton).^21^, ^22^

Recent studies have demonstrated that Shh stimulates embryonic stem cell proliferation via Gli family activation and protein kinase C cooperation in mice.^23^ The Hh signalling pathway also regulates the self‐renewal of mammary stem cells via Bmi1, a Polycomb group protein.^24^, ^25^ Bmi1 participates in brain development and stem cell proliferation. It is also able to replace some reprogramming factors such as Sox2, Klf4 and N‐myc when induced pluripotent stem (iPS) cells are generated from murine embryonic fibroblasts.^26^, ^27^ Synthetic or natural small molecules are widely used to understand and regulate stem cells.^20^ Small molecules such as purmorphamine and oxysterol activate the Shh signalling pathway. They are able to replace Bmi1 to generate iPS cells. They also induce Bmi1, Sox2 and N‐myc expression to promote the proliferation of neural precursor cells.^26^

Both the epithelial‐mesenchymal transition (EMT) and the mesenchymal‐epithelial transition (MET) play important roles in embryonic development, fibrosis and cancer progression.^28^, ^29^, ^30^ The EMT influences organ and tissue formation during embryogenesis, including the neural crest, heart, nervous system and craniofacial structure.^29^ More recently, the effect of the EMT on the self‐renewal and stemness of cancer stem cells (stem‐like cells in tumours) was studied.^31^ The EMT is characterized by cells losing their epithelial state and acquiring fibroblast‐like properties. Cells produced by the EMT show decreased intercellular adhesion and elevated motility.^25^ Specifically, they show decreased expression of E‐cadherin and increased expression of mesenchymal cell markers (such as N‐cadherin, fibronectin, vimentin and α‐smooth muscle actin.^28^, ^32^, ^33^

In the present study, the effects of the Shh signalling pathway on the generation and propagation of murine SKPs (mSKPs) were investigated. We also explored whether activation of the Shh signalling pathway contributes to the self‐renewal and cell proliferation of SKPs. The sphere formation of SKPs in suspension culture revealed a correlation between the EMT and the Shh signalling pathway. Our study highlights the fact that the Shh pathway is important to the self‐renewal and cell proliferation of SKPs.

## 2 MATERIALS AND METHODS

### 2.1 Animals

A total of 90 pregnant ICR female mice (DBL, Chungbuk, Korea) were used in our studies (8‐15 mice per group). Six to 15 embryos could be obtained from each pregnant female. All animal experiments were performed under the guidelines of the Institutional Animal Care and Use Committee of Seoul National University (approval number: SNU‐131231‐4).

### 2.2 Chemicals

All inorganic and organic compounds were obtained from Sigma‐Aldrich Korea (Yong‐in, Korea). All liquid solutions were purchased from Thermo Fisher Scientific Korea (Seoul, Korea) unless otherwise stated.

### 2.3 Propagation and isolation of mSKPs

The mSKPs were isolated by previously described protocols^1^, ^2^, with a few modifications. Back skin obtained from E16.5‐17.5 mouse embryos was washed three times in phosphate‐buffered saline (PBS; WelGENE, Daegu, Korea) with 3X penicillin/streptomycin (Gibco, Grand Island, NY, USA) and then minced into small pieces using a blade. Small pieces of back skin were incubated for 40 minutes in a 37°C, 5% CO_2_ cell culture incubator on a 60 mm culture dish containing 4 mL of 0.05% (w/v) trypsin solution (Gibco) or 1X TryPLE™ (Gibco). The incubated skin pieces were pipetted up and down 30 times for single cell dissociation. Dulbecco's modified Eagle medium (DMEM; WelGENE) with 10% foetal bovine serum (Atlas Biologicals, Fort Collins, CO, USA) was added onto the incubated skin pieces and dissociated single cells for enzyme neutralization. Skin and cell suspensions were strained through 100 and 40 μm nylon cell strainers (BD, Franklin Lakes, NJ, USA) for single cell isolation. Strained single cells were centrifuged at 250 ***g*** for 4 minutes. The cell pellet was resuspended in 5 mL of DMEM/F‐12 (3:1 mixture, v/v; Gibco) containing 2% B27 supplement (B27; Gibco), 20 ng/mL epidermal growth factor (EGF; Peprotech, Rocky Hill, NJ, USA), and 40 ng/mL basic fibroblast growth factor (bFGF; Peprotech; called SKP medium). The single cells were counted and then cultured in a 90 mm Petri dish in a 37°C with 5% CO_2_ atmosphere. Fresh SKP medium was replaced every 3 days. The cells were passaged every 5‐7 days. The formed spheres were single‐cell dissociated by pipetting with Accutase™ (Gibco).

### 2.4 Differentiation of mSKPs into various lineages

For differentiation, spheres were centrifuged in basal SKP medium without growth factors. The spheres were dissociated using the same methods as in subculture. Dissociated single cells or spheres were seeded on the coated plate. For staining, differentiated cells were fixed with 4% formaldehyde at 4°C for 1 hour and then washed twice with PBS. The cells were then stained with Oil red O. For neural differentiation, mSKPs were attached on laminin‐ and PDL‐coated six‐well plates (BD). Cells were incubated in neurobasal medium™ (Gibco), including B27 and 0.5 mmol/L dibutyryl cAMP (Peprotech), for 14 days.

### 2.5 Immunofluorescence staining

Immunofluorescence staining was performed according to standard protocols. Briefly, mSKPs or differentiated cells from mSKPs were fixed in 4% paraformaldehyde, permeabilized with 0.25% Triton X‐100 (Sigma) and blocked with 1% goat serum in PBS. The fixed cells were stained with antibodies against Table 1. The treated cells were covered with SlowFade antifade reagent with DAPI (SlowFade^™^ Gold antifade with DAPI) for nuclear staining and covered with a glass coverslip. Images were captured with confocal microscopes (LSM800; Carl Zeiss, Oberkochen, Germany; FV‐300, Olympus, Tokyo, Japan).

**Table 1:** Antibodies and dilutions for immunofluorescence analysis

Antibody	Dilution	Catalogue number	Company
PAX6	1:50	PA1‐801	Thermo Fisher Scientific, Waltham, MA, USA
NGFR (p75NTR)	1:500	AB1554	Millipore, Darmstadt, Germany
MBP	1:100	sc‐13912	Santacruz, Dallas, TX, USA
S100b	1:100	sc‐7852	Santacruz
α‐Sma	1:500	ab32575	Abcam, Cambridge, UK
Chd2 (N‐cad)	1:100	NBP1‐48309	Novus Biologicals, Littleton, CO, USA
Anti‐mouse IgG‐FITC	1:1000	A‐11029	Thermo Fisher Scientific
Anti‐rabbit IgG‐FITC	1:500	AP132F	Millipore
Anti‐goat IgG‐FITC	1:500	A‐11055	Thermo Fisher Scientific

### 2.6 Reverse transcription‐PCR

For total RNA isolation from sphere‐forming mSKPs, we followed the commercial protocol of the Ambion PureLink™ RNA Mini Kit (Thermo Fisher Scientific). For the synthesis of first‐strand cDNA, reverse transcription was performed for 1 hour at 42°C in a final reaction volume of 25 μL using cDNA synthesis kit (Thermo Fisher Scientific). Synthetic cDNA from the RNA of mSKPs and differentiated cells was used for each PCR reaction. Each reaction contained 50 ng cDNA, 20 pmol each of specific primers, and AccuPower™ PCR Premix (Bioneer, Daejeon, Korea). Thermal cycle was repeated 34 times.

### 2.7 Real‐time quantitative PCR

The cDNA was analysed using real‐time quantitative PCR (qPCR). For optimal quantification, primers were designed using Primer Express software (Applied Biosystems, Foster City, CA, USA). The qPCR reactions were performed using the ABI PRISM 7500 system and SYBR™ Premix Ex Taq II (Takara Bio Inc., Shiga, Japan). All samples were run in triplicate as technical replicates. The following amplification procedure was employed. Data were analysed using the 7500 System Sequence Detection software (Applied Biosystems). All samples had the same starting quantities of all candidate reference genes, based on the standard curves generated for those genes. All procedures and data analyses followed MIQE guidelines.^34^ The specific primer sequences targeting genes for stemness, differentiation, EMT, Shh signalling and the neural crest are listed in Table 2.

**Table 2:** Primer sequences for qPCR analysis

Gene	Primer sequence (5′ to 3′)		Accession
CD49f	Forward	GATGCTGCCAACGCTGTATTC	NM_001277970
	Reverse	GCCGTTCTGGCAACAGATG	
c‐Myc	Forward	TGCGGTCGCTACGTCCTT	NM_010849
	Reverse	TCCAAGTAACTCGGTCATCATCTC	
Oct4	Forward	CCGTGTGAGGTGGAGTCTGGAG	NM_013633
	Reverse	GCGATGTGAGTGATCTGCTGTAGG	
NGFR	Forward	CAGGGAAACATCTGGAAACGA	NM_033217
	Reverse	TGGACCAGGTTTTGAACAGACA	
Smo	Forward	AAGGCCACCCTGCTCATCTG	NM_176996
	Reverse	AGGCCTTGGCGATCATCTTG	
Nanog	Forward	AGGACAGGTTTCAGAAGCAGAAGT	NM_028016
	Reverse	TCAGACCATTGCTAGTCTTCAACC	
Klf4	Forward	ACTATGCAGGCTGTGGCAAAA	NM_01063
	Reverse	CCGTCCCAGTCACAGTGGTA	
Bmi1	Forward	CGCTCTTTCCGGGATCTTTT	NM_007552
	Reverse	CCCTCCACACAGGACACACA	
Twist	Forward	AGAAGAGCAGAGACCAAATTCACA	NM_011658
	Reverse	GCTGCCCCTCTGGGAATC	
Nestin	Forward	GGCATCCCTGAATTACCCAA	NM_016701
	Reverse	AGCTCATGGGCATCTGTCAA	
Lsd1	Forward	GTTCATCAGGAATCGCACATTG	NM_001347221
	Reverse	GCTGTTGTAAGGCGCTTCCA	
Sox9	Forward	GCAGACCAGTACCCGCATCT	NM_011448
	Reverse	CCTCCACGAAGGGTCTCTTCT	
Patched1	Forward	CGAGACAAGCCCATCGACATTA	NM_001328514
	Reverse	AGGGTCGTTGCTGACCCAAG	
Cdh2	Forward	AGCGCAGTCTTACCGAAGG	NM_007664
	Reverse	TCGCTGCTTTCATACTGAACTTT	
Fn1	Forward	GATGTCCGAACAGCTATTTACCA	NM_001276408
	Reverse	CCTTGCGACTTCAGCCACT	
Vim	Forward	CGTCCACACGCACCTACAG	NM_011701
	Reverse	GGGGGATGAGGAATAGAGGCT	
Tgfb1	Forward	CTCCCGTGGCTTCTAGTGC	NM_011577
	Reverse	GCCTTAGTTTGGACAGGATCTG	
a‐Sma	Forward	GTC CCA GAC ATC AGG GAG TAA	NM_007392
	Reverse	TCG GAT ACT TCA GCG TCA GGA	
Gapdh	Forward	AGGTCGGTGTGAACGGATTTG	NM_001289726
	Reverse	TGTAGACCATGTAGTTGAGGTCA	

### 2.8 Protein extraction and Western blot

The mSKPs receiving various treatments were centrifuged and collected. Collected mSKPs were lysed by Passive lysis buffer (Promega, Madison, WI, USA). Protein was quantified using the Pierce BCA protein assay kit (Thermo Fisher Scientific). Equal amounts of protein (30‐50 μg) from each treated group were analysed on a 12% sodium dodecyl sulphate polyacrylamide gel. After transfer to a nitrocellulose membrane, the membrane was incubated with primary and secondary antibodies on a shaker. Detection was performed using WesternBright™ Quantum (Advansta, Menlo Park, CA, USA), according to the manufacturer's recommended protocol. Western blot data were analysed using the GeneGnome XRQ System (Syngene, Cambridge, UK).

### 2.9 Cell proliferation assays

The mSKPs were measured for cell proliferation using the WST‐1 cell proliferation assay (Daeil Lab Service, Seoul, Korea), following the manufacturer's instructions. Briefly, mSKP spheres were harvested by centrifugation. The mSKPs were dissociated to single cells by accutase. Dissociated cells were serially diluted and then plated on 96‐well plates in SKP medium. Each plate was analysed by a macrowell reader at an absorbance of 450 nm, after plated cells had been cultured for 48‐72 h.

### 2.10 Apoptosis analysis by Annexin V assay using fluorescence‐activated cell sorting

The mSKPs were plated at a density of 1.5 × 10^5^ cells/ml. Cultured suspension cells were treated with the small molecules Pur, CP and/or GANT‐61 for 72 hours. Cells were stained with an Annexin V assay kit (Cayman, Ann Arbor, MI, USA) according to the manufacturer's protocol. Briefly, SKPs were dissociated by accutase and then washed twice in cold PBS. Cells were resuspended in binding buffer, and 10 ml of FITC‐conjugated Annexin V was added. PI or 7‐AAD was also added to detect nonviable cells. Dissociated cells were incubated for 15 minutes in the dark, and an additional 400 mL of binding buffer was added. The cells were analysed within 1 hour by flow cytometry. Acquisition was performed on a FACS Calibur instrument using CellQuest Pro software (BD). Each analysis was performed on at least 10 000 events.

### 2.11 Statistical analyses

All numerical values in this study are expressed as the mean ± SD. Statistical analyses were performed using a two‐tailed Student's *t* test for comparison between two groups, or a one‐way ANOVA for the comparison of three or more groups. Differences were considered statistically significant at *P* values <0.05.

## 3 RESULTS

### 3.1 Isolation, culture and characterization of mSKPs from murine foetal back skin

**Figure 1:**
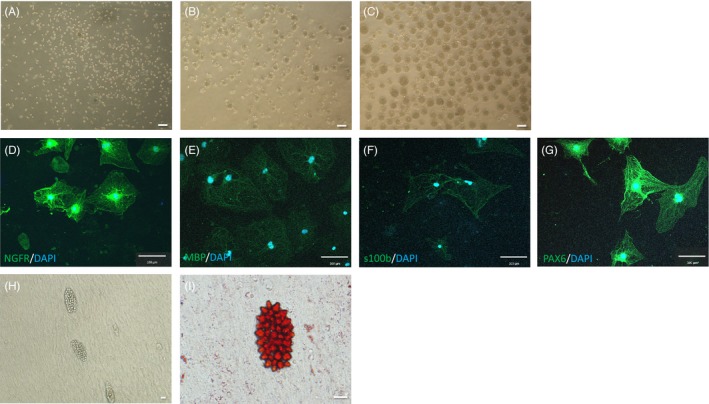
Isolation and differentiation of murine skin‐derived precursors (mSKPs). (A) Single cells were separated from the back skin of murine foetuses. (B) Primary spheres were generated from isolated single cells. (C) Secondary spheres were larger and more condensed than primary spheres. (D‐G) The mSKPs were differentiated into Schwann cells on PDL and laminin‐coated dishes from d 14 to 21, using Schwann cell differentiation medium. The differentiated cells exhibited immunofluorescence (green) for (D) nerve growth factor receptor (NGFR), (E) myelin basic protein (MBP), (F) S100 calcium‐binding protein (S100B) and (G) neural crest marker PAX6. DAPI (blue) is shown in merged images (D‐G). (H, I) The mSKPs were differentiated into adipogenic cells. The differentiated adipogenic cells from mSKPs were stained by Oil Red O. Scale bars: 100 μm

The mSKPs formed spheres from single cells in suspension culture (Figure 1A). After 5‐7 days, the spheres grew larger, and some spheres began to aggregate. The mSKPs proliferated, expanded and reformed spheres after subculture via accutase and pipetting (Figure 1B,C). After 3‐4 weeks of neural differentiation induction, neural cells were identified by immunofluorescence analysis using neural cell markers that are only expressed in differentiated cells, such as NGFR, MBP, s100b and PAX6 (Figure 1D‐G). The mSKP spheres also differentiated into adipogenic cells (Figure 1H). The differentiated adipogenic cells were confirmed by Oil Red O (Figure 1I). These results demonstrate that cell spheres isolated from murine back skin have SKP properties.

### 3.2 Activation of the Shh pathway by recombinant Shh (rShh) treatment in mSKPs

**Figure 2:**
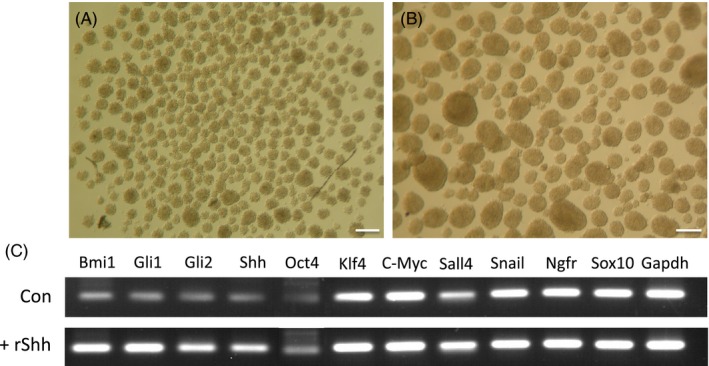
The effect of recombinant Shh (rShh) on mSKPs. The morphology of expanded mSKP spheres; (A) untreated control. (B) After rShh treatment. (C) RT‐PCR analysis of gene expression in mSKPs after rShh treatment. Scale bars: 100 μm

After treatment of 500 ng/mL rShh, mSKPs in the treated group formed larger spheres than the control group (Figure 2A,B). Several genes related to stemness, the neural crest and the Shh pathway were detected in the reverse transcription‐PCR analysis (Figure 2C). These findings indicate that sphere formation and multipotency in mSKPs are influenced by activation of the Shh signalling pathway.

### 3.3 Treatment with a Shh agonist promotes the proliferation of mSKPs and changes gene expression

**Figure 3:**
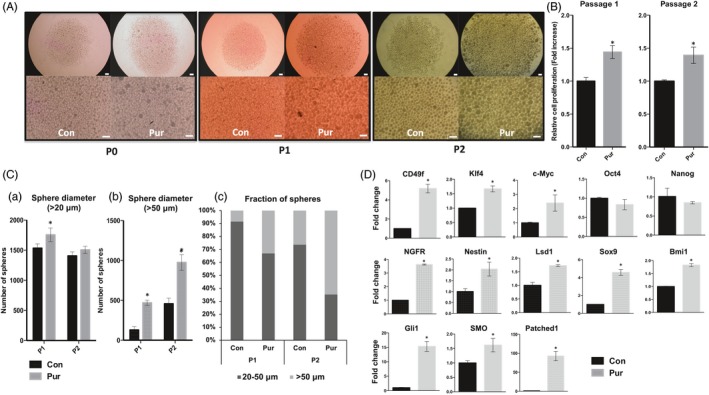
The effect of purmorphamine (Pur) on mSKPs. (A) mSKP spheres formed at passage 0 (P0), P1 or P2 after Pur treatment. (B) The cell proliferation of mSKPs after Pur treatment was examined by WST‐1 assay at P1 and P2. (C) The sphere‐forming efficiency of mSKPs was measured. The number of spheres with a diameter more than (a) 20 μm or (b) 50 μm was compared between the control and Pur‐treated groups. (c) The relative abundance of large (>50 μm) and small (>20 μm) spheres was measured at P1 and P2. (D) The mRNA expression of Oct4, Nanog, c‐Myc, Klf4, N‐cad, Twist, Ngfr, Smo, nestin, Lsd1, Bmi1, Sox9, Patched1 and Gli1 was measured by qPCR in mSKPs. Values were normalized against GAPDH and depicted as fold‐change values relative to the control (no Pur treatment; control value = 1). *P <* *0.05. Scale bars: 100 μm

The mSKPs treated with Pur (a Shh agonist) formed larger spheres, and their number also increased compared to the control group in all passages checked (Figure 3A). The results of a WST‐1 assay showed a significant increase in the proliferation rate after treatment with Pur at passages 1 and 2 (Figure 3B). Although the total number of spheres with a diameter over 20 μm was not different from the control group (Figure 3C‐a), the number of spheres with a diameter over 50 μm increased 5.8‐ and 2.5‐fold with Pur treatment at passages 1 and 2, respectively (Figure 3C‐b). After Pur treatment, the number of mSKPs at passage 2 was twofold higher than at passage 1 (Figure 3C‐c). These results show that activation of the Shh signalling pathway by its agonist increases proliferation and sphere formation in mSKPs. A potential critical task of the Shh pathway is the self‐renewal of mSKPs.

Treatment with Pur increased the expression of the following genes: c‐Myc and Klf4 (reprogramming factors); NGFR and nestin (neural progenitor‐related markers); CD49f, Sox9 and Bmi1 (stem cell markers); and Smo, Gli1 and Patched1 (Shh pathway‐related makers). Thus, Pur promotes self‐renewal and stemness in mSKPs by increasing the expression of stem cell‐, progenitor‐ and reprogramming‐related genes via Shh signalling pathway activation.

### 3.4 Treatment with a Smo inhibitor decreases proliferation in mSKPs and changes gene expression

**Figure 4:**
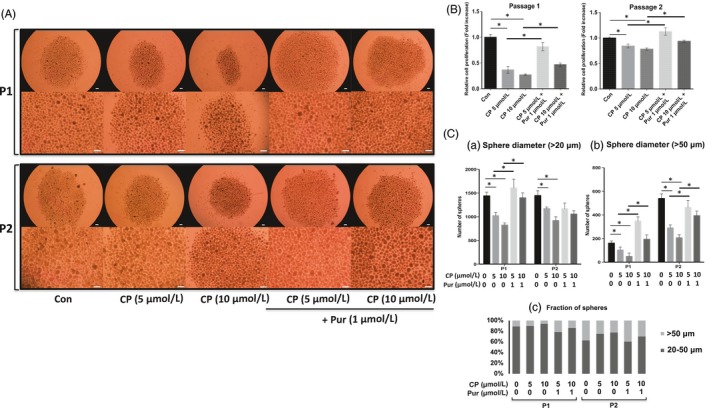
The effect of cyclopamine (CP) on mSKPs. (A) The mSKP spheres formed in SKP medium at P1 or P2 after combined treatment with CP (5 or 10 μmol/L) and Pur. (B) Cell proliferation of the mSKPs at P1 and P2 was assessed by WST‐1 assay after combined treatment with CP and Pur. (C) The sphere‐forming efficiency of mSKPs was measured to confirm the inhibition of Shh signalling pathway by CP and counteraction by Pur in CP‐mediated inhibition. The number of spheres with a diameter more than (a) 20 μm or (b) 50 μm was compared between the control, Pur and CP‐treated groups. (c) The relative abundance of large (>50 μm) and small (>20 μm) spheres was measured at P1 and P2 for each treated group. *P <* *0.05. Scale bars: 100 μm

The size and number of spheres decreased after treatment with CP (a Smo inhibitor), as observed using a stereomicroscope (Figure 4A,S1). A dose‐dependent decrease in the proliferation of mSKPs due to CP was also demonstrated by a WST‐1 assay (Figure 4B). Cell morphology and proliferation in CP‐ and Pur‐treated mSKPs were similar to the control group (Figure 4A,B).

CP treatment decreased the total number of spheres, regardless of their diameter. The number of spheres after cotreatment with CP and Pur was similar to the controls, regardless of diameter. CP‐mediated effect was counteracted by Pur treatment (Figure 4C‐a,b). In addition, the effect of counteraction by cotreatment on the proliferation and sphere formation of murine SKPs was dependent on the dose of CP (Figure 4B,C). These results demonstrate that Shh inhibition decreases the formation and proliferation of cellular spheres (Figure 4C‐c).

**Figure 5:**
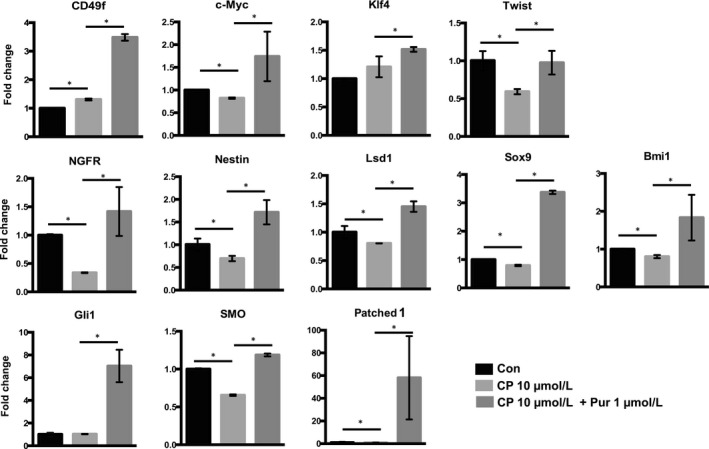
qPCR analyses of mRNA expression in CP‐treated mSKPs. The mRNA expression of Oct4, Nanog, c‐Myc, Klf4, N‐cad, Twist, NGFR, Smo, Nestin, Lsd1, Bmi1, Sox9, Patehed1 and Gli1 was measured in CP‐ and Pur‐treated mSKPs. Values were normalized against GAPDH and depicted as fold‐change values relative to the control (no CP and Pur treatment; control value = 1). *P <* *0.05

The mRNA levels of the markers were analysed in 10 μmol/L CP‐treated SKPs which were most effective condition in the proliferation and sphere formation. Treatment with CP decreased the expression of Gli1 and Smo (Shh pathway genes) and nestin and Ngfr (neural stem cell/precursor genes). However, the expression of pluripotency and reprogramming genes such as Oct4, Nanog, c‐Myc and Klf4 was not significantly different between the CP‐treated and control groups. In addition, Pur treatment elevated the expression of several key genes which had been decreased by CP treatment (Figure 5). Therefore, it is possible that Pur treatment may recover a CP‐inhibited Shh signalling pathway.

### 3.5 The effect of a Gli inhibitor (GANT‐61) on the self‐renewal and proliferation of mSKPs

**Figure 6:**
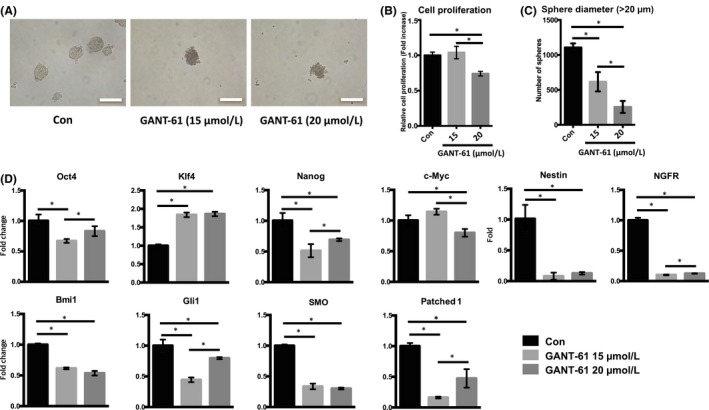
The effect of GANT‐61 on mSKPs. (A) The mSKP spheres formed at P2 after GANT‐61 (15 or 20 μM) treatment for 72 h. (B) The cell proliferation of mSKPs was examined by WST‐1 assay after GANT‐61 treatment. (C) The number of spheres with a diameter more than 20 μm was compared between the control and GANT‐61 (15 or 20 μmol/L) treatment group. (D) The mRNA expression of Nanog, Ngfr, nestin, Bmi1, Patehed1 and Gli1 was measured by qPCR after GANT‐61 treatment. Values were normalized against GAPDH and depicted as fold‐change values relative to the control (no GANT‐61 treatment; control value = 1). *P <* *0.05. Scale bars: 100 μm

The influence of the Shh‐Gli signalling pathway on mSKPs was investigated using the Gli inhibitor GANT‐61. Treatment of mSKPs with GANT‐61 led to reduced sphere formation compared to the control (Figure 6A,S2A), and cell proliferation also decreased at passages 1 and 2 (Figure 6B,S2B). Sphere size and number were also reduced by Gli1 inhibition (Figure 6C). In addition, the expression of genes related to stemness and Shh signalling was decreased by the Gli1 inhibitor (Figure 6D). These results demonstrated that the formation and proliferation of mSKP spheres are inhibited by a block of the Shh‐Gli1 signalling pathway, caused by a Gli1 inhibitor.

### 3.6 Treatment with CP or GANT‐61 influences apoptosis in mSKPs

**Figure 7:**
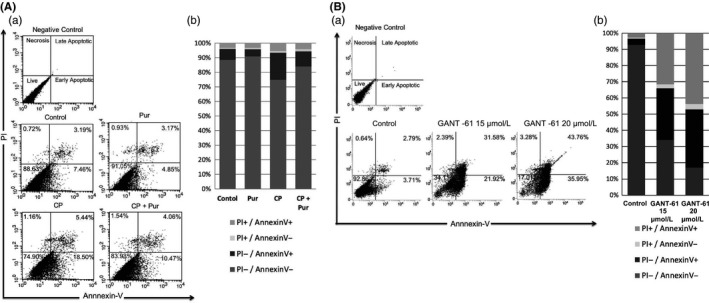
Apoptosis of mSKPs after treatment with CP or GANT‐61. Apoptotic cells were analysed by FACS in (A) CP‐ and Pur‐treated mSKPs, or (B) GANT‐61 (15 or 20 μmol/L)‐treated mSKPs. Representative biparametric FACS data are derived from combined PI‐ and FITC‐conjugated Annexin V staining. Percentage of PI+/Annexin V‐ (top left), PI+/Annexin V+ (top right), PI‐/Annexin V+ (bottom right) and PI‐/Annexin V‐ (bottom left). (Aa, Ba) Percentage of sorted cells in each quadrant. (Ab, Bb) FACS data represented by a 100% stacked column chart. n = 4

CP treatment increased apoptosis in mSKPs. In particular, CP induced early apoptosis and resulted in a decrease of live cells. Co‐treatment with Pur and CP increased live cells and yielded fewer early apoptotic cells (Figure 7A). GANT‐61 also decreased live cells and induced both early and late apoptosis. It did this dramatically, in a dose‐dependent manner via Gli1 inhibition (Figure 7B). The data demonstrate that Smo or Gli1 inhibition by CP or GANT‐61 induces apoptosis and inhibits sphere formation. This result shows that the Shh signalling pathway directly regulates the self‐renewal of mSKPs.

### 3.7 The Shh signalling pathway regulates the EMT in mSKPs

**Figure 8:**
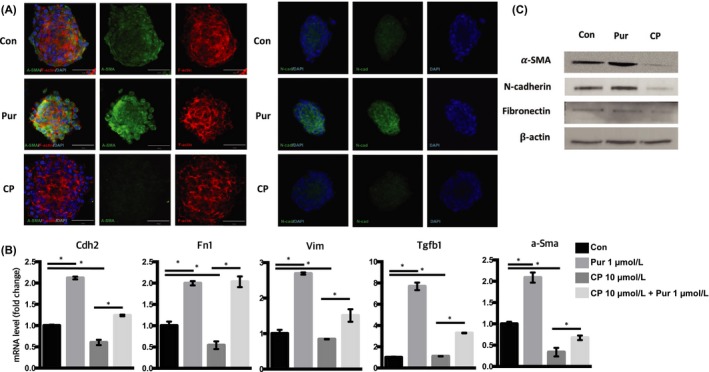
Regulation of epithelial‐mesenchymal transition (EMT) genes by the Shh signalling pathway. (A) The mSKP spheres after CP or Pur treatment were stained with α‐SMA or N‐cadherin (N‐cad; green), and with F‐actin (red). DAPI (blue) indicates nuclei. (B) The mRNA expression of α‐Sma, Cdh2 (N‐cad), vimentin (Vim), fibronectin (Fn1) and Tgf‐β1 was measured by qPCR in CP‐treated mSKPs (with or without Pur) at 72 h. (C) The relative protein levels of α‐SMA, N‐cad and fibronectin were analysed by Western blot analysis. The housekeeping protein β‐actin was used to control for loading. *P <* *0.05. Scale bars: 50 μm

To examine the correlation between the Shh signalling pathway and the EMT in mSKPs, expression levels of EMT markers (such as α‐Sma, Cdh2, Fn1, Vim and Tgf‐β1) were compared between Pur‐treated and nontreated mSKPs. The expression of EMT markers was increased by Shh activation, whereas expression was decreased by Shh inhibition. In addition, Pur recovered EMT markers that were inhibited by CP treatment (Figure 8A). The Pur‐promoted activation of the Shh signalling pathway elevated EMT protein levels, as measured by Western blot and immunofluorescence. In contrast, inhibition of the Shh signalling pathway by CP decreased EMT protein expression. Among the EMT genes, α‐Sma and Cdh2 were strongly affected by the Pur and CP treatments (Figure 8B,C). The result shows that the Shh signalling pathway also regulates the EMT phenotype in mSKPs.

### 3.8 Using Pur to promote activation of the Shh signalling pathway for the long‐term culture of mSKPs

**Figure 9:**
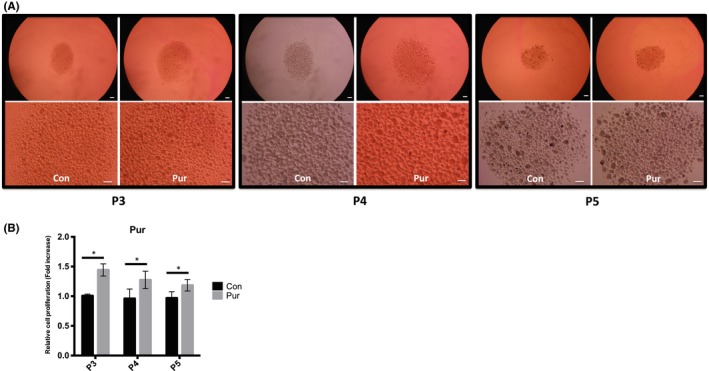
The effect of Pur treatment during long‐term culture of mSKPs. (A) The mSKP spheres formed at P3, P4 and P5 after Pur treatment. (B) The cell proliferation of mSKPs was examined by WST‐1 assay after Pur treatment. *P <* *0.05. Scale bars: 100 μm

Although sphere formation in mSKPs was remarkably decreased after passage 3, proliferation and sphere formation in mSKPs were improved by Pur treatment (Figure 9A,B). These data suggest that activation of the Shh signalling pathway by Pur can revive the self‐renewal and proliferation of aged mSKPs.

## 4 DISCUSSION

The objective of this study was to investigate sphere formation and cell proliferation using Pur, a Shh agonist, to activate the Shh signalling pathway. We demonstrated that Pur increases the expression of stem cell genes (CD49f, Ngfr, nestin, Klf4 and Bmi1) and EMT genes (N‐cad, α‐Sma, fibronectin, vimentin and Tgf‐β1) in mSKPs. Our findings suggest that Pur promotes the proliferation of mSKPs in culture, and that the Shh signalling pathway regulates the self‐renewal of mSKPs.

The Hh signalling pathway is involved in the survival, proliferation and differentiation of cells in embryonic development.^20^, ^21^, ^35^ Many other studies have shown that aberrant signalling in this pathway is related to a variety of human cancers. These include basal cell carcinomas, colorectal cancer, ovarian cancer and small‐cell lung cancer.^14^, ^29^, ^30^, ^36^ Activation of the Shh signalling pathway has an essential role in controlling self‐renewal and tumour initiation in melanoma.^37^ In addition, the Shh signalling pathway increases the initial generation and self‐renewal of neural cells.^16^ To our knowledge, there is no available study as to whether the Shh signalling pathway influences the proliferation, self‐renewal and apoptosis of mSKPs. We demonstrated that Pur treatment enhances the sphere formation capability of mSKPs, and this result shows that the Shh signalling pathway is related to the self‐renewal and proliferation of mSKPs.

It has been suggested that Hh signalling plays a critical role in regulating the proliferation of various types of stem cells, including mammary, telencephalic and mesenchymal stem cells. Pur enhances cell proliferation and reduces apoptosis in human umbilical cord blood‐derived mesenchymal stem cells. This is achieved through the RNA‐binding protein Msi1, which regulates oncogenes, cell cycle genes and microRNAs.^38^ We used Pur to activate the Shh signalling pathway because Pur showed a similar effect to rShh. After Pur treatment, mSKPs were evaluated according to sphere size and number to verify the capacity for sphere formation. It has been reported that the PI3K‐AKT signalling pathway promotes self‐renewal and inhibits senescence in human SKPs treated with small molecules.^13^ We confirmed that the number of spheres with a diameter longer than 50 μm increased at passages 1 and 2 after Pur treatment. Our results suggest that Pur treatment activates the Shh signalling pathway to promote cell proliferation and self‐renewal in SKPs. Although the expression of key pluripotency genes (Oct4 and Nanog) did not change with Pur treatment, neural and adult stem cell markers (nestin, CD49f, Klf4, and Ngfr) increased, indicating the stemness‐enhancing property of Pur.

Shh regulates the self‐renewal of stem cells through canonical and noncanonical hedgehog pathways that are related to the Smo receptor.^23^, ^39^ The chemical regulation of Hh signalling may have biomedical applications, such as the treatment of Hh signalling pathway‐related cancers or the differentiation of stem cells.^31^, ^40^, ^41^ The secreted Hh protein binds to Patched1, which represses the activity of Smo in the Hh signalling pathway. This promotes the expression of Hh target genes by affecting Gli family transcription factors.^30^, ^42^ Pur also modulates Smo activity through binding to Patched1.^43^ Our data show that Pur specifically activates the Shh signalling pathway by increasing the expression of Patched1 and Gli1.

The Shh signalling pathway can be inhibited by various small molecules that target disparate members of the pathway.^22^ In particular, the Smo antagonist CP acts by binding to Smo. CP treatment reduces the proliferation of hippocampal neural progenitor cells in vivo and in vitro and cancer cells in vitro.^15^, ^16^, ^43^ In our study, sphere proliferation and the number of spheres were reduced by CP treatment in a dose‐dependent manner. This result implies that inhibition of the Shh signalling pathway by a Smo antagonist depresses the self‐renewal of mSKPs. However, stemness‐related genes such as Klf4, CD49f and c‐Myc did not decrease with the Shh signalling pathway inhibition caused by CP. Although proliferation and clonogenicity in human mesenchymal stem cells decrease with inhibition of the Hh signalling pathway, this does not influence their differentiation potential.^39^ Recent studies have shown that CP induces apoptosis and inhibits the proliferation of cancer cells and cancer stem cells.^31^ Our findings after CP treatment suggest that the Shh signalling pathway is important to the apoptosis of mSKPs.

In the present study, the Shh signalling pathway was directly inhibited by GANT‐61, a Gli1 inhibitor. Gli1 is an essential gene in the Hh signalling pathway and plays an important role in tumour progression.^32^, ^36^ The Shh‐Gli signalling pathway is abnormally active in certain cancers, and inhibition of Gli function is important to tumour therapy.^36^, ^42^ In normal tissues, Gli is primarily active in precursor cells. The direct inhibition of Gli1 dramatically induces apoptosis in cancer stem cells and tumour cells.^14^, ^32^, ^42^ However, the role of Gli1 in SKPs and stem cells is not clear. GANT‐61, which directly inhibits Gli, was used to investigate the effect of Gli1 on proliferation, self‐renewal and apoptosis in mSKPs. The ability of GANT‐61 to block the Hh‐Gli pathway has been reported in many preclinical and basic studies.^17^, ^30^ GANT‐61 primarily represses self‐renewal in cancer cells via inhibition of the Shh signalling pathway.^31^, ^32^, ^37^ Our data suggest that GANT‐61 causes abnormal spheroid shape formation in vitro. Furthermore, apoptosis increased significantly with GANT‐61 treatment. These findings imply that the Shh‐Gli pathway is critical to self‐renewal in mSKPs and acts by regulating apoptosis.

Changes in EMT gene expression after activation of the Shh signalling pathway were investigated. The Shh signalling pathway affects the EMT, especially during embryonic development and during metastasis in various cancers.^28^, ^30^ Self‐renewals in pancreatic cancer stem cells decreased with inhibition of the Shh signalling pathway by sulphoraphane.^32^ Our results show that certain EMT genes (N‐cad, α‐Sma, vimentin, fibronectin and Tgf‐β1) increased during sphere formation and propagation when Shh was activated by Pur. This increase in EMT gene expression contributes to the self‐renewal and proliferation of SKPs, similar to the case in stem cells.^28^, ^32^ EMT gene expression decreased after the Shh signalling pathway was inhibited by CP or GANT‐61 treatment. These data suggest that the Shh signalling pathway and the EMT are associated with self‐renewal and proliferation during sphere formation in mSKPs.

When human and mouse SKPs are cultured long‐term, ageing and senescence occur. Sphere formation and cell proliferation are reduced, and these cells cannot maintain their self‐renewal potency at late passages.^13^ Our results show that activation of the Shh signalling pathway by Pur treatment improves the self‐renewal of mSKPs during long‐term culture in vitro.

In conclusion, the Shh‐Gli signalling pathway plays an important role in the self‐renewal, proliferation and inhibition of apoptosis in mSKPs. Pur is critical for the expansion of mSKPs since it enhances self‐renewal and proliferation by activating the Shh signalling pathway. In the future, human SKPs could possibly be grown to sufficient numbers for therapy. The results of this study provide fruitful information that adds to our knowledge of stem cells and skin development.

## ACKNOWLEDGEMENT

This study was supported by a grant from the National Research Foundation of Korea (NRF‐2016R1D1A1B03931864) and by the Technology Development Program (S2423830) funded by the Ministry of SMEs and Startups (MSS, Korea).

Study design: Sangkyu Park. Data collection: Sangkyu Park, Haewon Kim, Kichul Kim. Data analysis: Sangkyu Park, Haewon Kim, Kichul Kim. Manuscript preparation: Sangkyu Park, Sangho Roh.

## CONFLICT OF INTEREST

The authors declare no conflict of interest.
