# Supplementary figures and images for: Sonic hedgehog signalling regulates the self‐renewal and proliferation of skin‐derived precursor cells in mice
Source: Cell Prolif. 2018 Aug 27;51(6):e12500. doi: 10.1111/cpr.12500 (PMC6528853; doi:10.1111/cpr.12500)

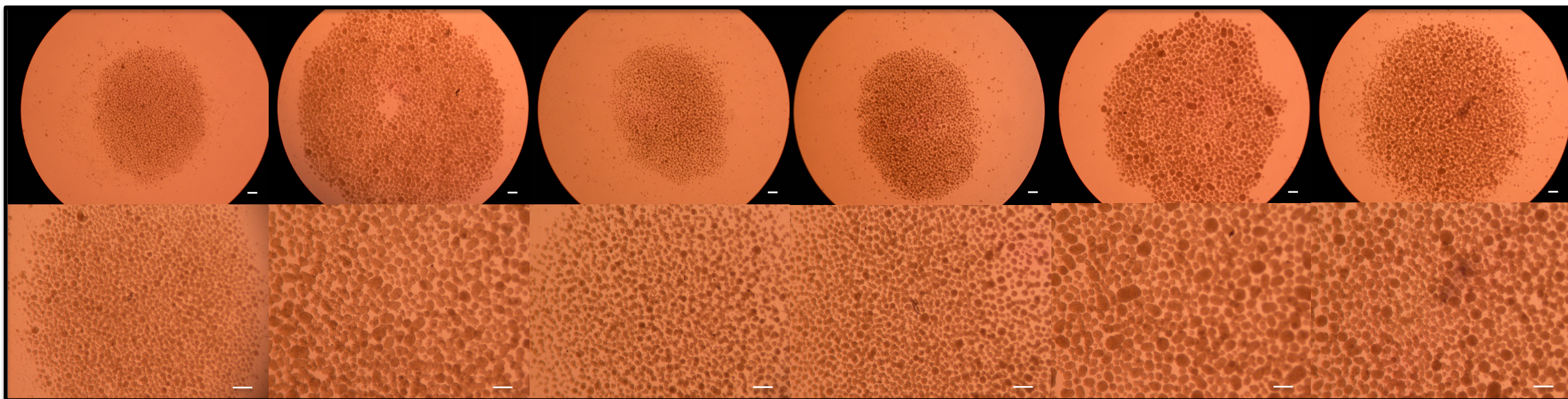

Con

Pur (1  $\mu$ M)

CP (5  $\mu$ M)

CP (10  $\mu$ M)

CP (5  $\mu$ M)

CP (10  $\mu$ M)

+ Pur (1  $\mu$ M)

Supplement: Supplementary file 1 [file CPR-51-na-s001.pdf]

(A)

P1

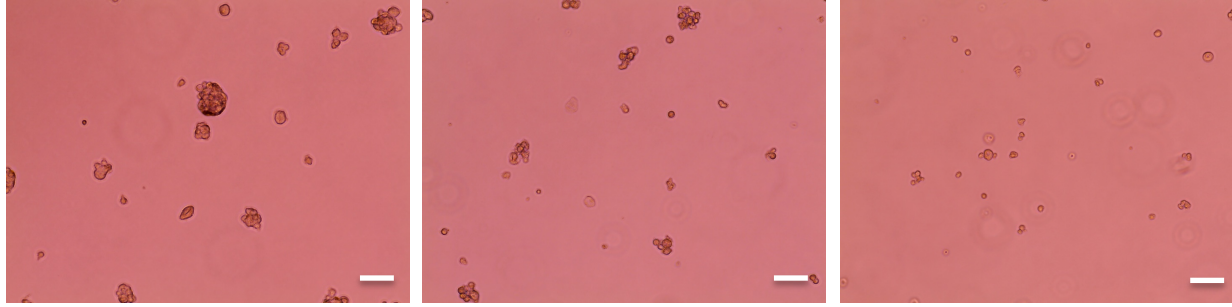

Con

GANT-61 (15 μM)

GANT-61 (20 μM)

(B)

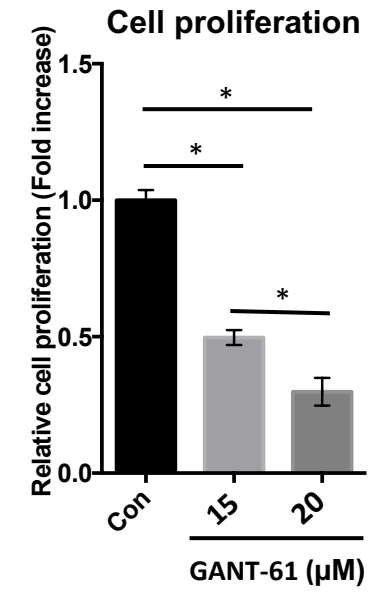

Supplement: Supplementary file 2 [file CPR-51-na-s002.pdf]
